# Prognosis of neonatal tetanus in the modern management era: an observational study in 107 Vietnamese infants

**DOI:** 10.1016/j.ijid.2014.12.011

**Published:** 2015-04

**Authors:** Phung Khanh Lam, Huynh T. Trieu, Inke Nadia D. Lubis, Huynh T. Loan, Tran Thi Diem Thuy, Bridget Wills, Christopher M. Parry, Nicholas P.J. Day, Phan T. Qui, Lam Minh Yen, C. Louise Thwaites

**Affiliations:** a Oxford University Clinical Research Unit, Hospital for Tropical Diseases, 764, Vo Van Kiet, Ho Chi Minh City, Vietnam; b Hospital for Tropical Diseases, Ho Chi Minh City, Vietnam; c Centre for Tropical Medicine and Global Health, Nuffield Department of Medicine, University of Oxford, Oxford, UK; d University of North Sumatera, Medan, Indonesia; e Clinical Sciences, Liverpool School of Tropical Medicine, Liverpool, UK; f Mahidol Oxford Tropical Medicine Research Unit, Faculty of Tropical Medicine, Mahidol University, Bangkok, Thailand

**Keywords:** Neonatal tetanus, Management, Prognosis, Outcome

## Abstract

**Objectives:**

Most data regarding the prognosis in neonatal tetanus originate from regions where limited resources have historically impeded management. It is not known whether recent improvements in critical care facilities in many low- and middle-income countries have affected indicators of a poor prognosis in neonatal tetanus. We aimed to determine the factors associated with worse outcomes in a Vietnamese hospital with neonatal intensive care facilities.

**Methods:**

Data were collected from 107 cases of neonatal tetanus. Clinical features on admission were analyzed against mortality and a combined endpoint of ‘death or prolonged hospital stay’.

**Results:**

Multivariable analysis showed that only younger age (odds ratio (OR) for mortality 0.69, 95% confidence interval (CI) 0.48–0.98) and lower weight (OR for mortality 0.06, 95% CI 0.01–0.54) were significantly associated with both the combined endpoint and death. A shorter period of onset (OR 0.94, 95% CI 0.88–0.99), raised white cell count (OR 1.17, 95% CI 1.02–1.35), and time between first symptom and admission (OR 3.77, 95% CI 1.14–12.51) were also indicators of mortality.

**Conclusions:**

Risk factors for a poor outcome in neonatal tetanus in a setting with critical care facilities include younger age, lower weight, delay in admission, and leukocytosis.

## Highlights

• Large contemporary case-series in a setting of improved critical care facilities.
• Analysis of variables, some not analysed in previous studies/meta-analyses.
• Age and weight were associated with a poor outcome.
• Delay in admission to hospital and leukocytosis were also associated with a poor outcome.

## 1 Introduction

The worldwide incidence of neonatal tetanus has reduced significantly following a sustained initiative by the World Health Organization (WHO) and its partners The United Nations Children's Fund (UNICEF) and The United Nations Population Fund (UNFPA). Latest figures (June 2014) show only 24 countries out of 59 originally targeted have still to eliminate the disease.^1^ The initiative attempts to eliminate maternal and neonatal tetanus through improvements in maternal vaccination programmes, delivery practices, and surveillance systems. In some ‘high-risk’ areas, supplementary immunization programmes targeting all women of child-bearing age have been employed.^2^

Despite these advances, the disease continues to occur. Elimination of neonatal tetanus is defined as ‘less than one case per thousand live-births in every district of a country’.^3^ However, this does not mean complete eradication, as the causative agent of tetanus, *Clostridium tetani*, is ubiquitous in the environment throughout the world and is able to cause disease in any vulnerable individual (i.e., neonate with unvaccinated mother).

Maintaining a country's neonatal tetanus elimination status requires continued efforts and further resources to strengthen vaccination programmes, reproductive health services, and surveillance systems. These can be threatened by war or natural disasters. Tetanus clusters have been reported following tsunamis and earthquakes in Indonesia, Kashmir, and Haiti.^4^ It is still not clear how maternal HIV and malaria affect transplacental protective antibody transfer,^5,6^ although maternal HIV reduces the maternal response to tetanus vaccination.^7^

In neonatal tetanus, entry of tetanus toxin into the central nervous system results in muscle spasms, initially interfering with the ability to suck and feed, but later involving the chest muscles, impeding respiration. Without medical treatment mortality rates are very high (some reports are of 99% fatality).^8,9^ As tetanus evolves over days in a characteristic manner, the recognition of prognostic features on presentation may enable timely intervention and triage.

Generally, rapid disease progression in tetanus is associated with a worse outcome.^8,10^ Most studies have been performed in adults, but several case series in neonates have found similar indicators of prognosis.^11–13^ Lambo and Anokye recently performed a meta-analysis and included data from 4535 neonates to ascertain which features are most relevant in neonates. They concluded that low birth weight and age at onset were the most important factors in determining the outcome.^14^ The authors criticized the studies included in the analysis for limited prospective data and lack of control for gestational age. They noted that only one out of the 16 studies included all of the prognostic factors selected for the analysis and they were unable to find consistent reporting on delay in admission to hospital or duration of hospital stay to include these in their analysis, as originally intended. In some studies, current standard therapies such as tetanus anti-toxin were not necessarily used. Many of the studies included were conducted over 40 years ago, and even relatively recent studies used patient data from preceding decades. (The study by Patel and Mehta published in 1999 and including 1490 neonates, used data from the period 1954–68.^8^) In total, 3648 out of 4535 cases were admitted before 1996. Most of the studies were therefore either conducted in settings without facilities for mechanical ventilation or were performed before such facilities were readily available.

The availability of mechanical ventilation allows respiratory muscle spasm to be controlled and prevents respiratory failure – the major cause of death in tetanus. Recent improvements in critical care capacity in many low- and middle-income countries has meant improved supportive therapy is now available for a larger number of cases of neonatal tetanus. However, it is not known whether this has affected the reliability of factors identified to be associated with a worse prognosis in settings without these facilities.

In all settings the ability to rapidly identify those neonates at highest risk of a poor outcome may be especially important to target medical and nursing care appropriately. Knowledge of prognostic factors is also important to determine the efficacy of interventions over time and between locations. Baseline comparison of prognostic factors allows more accurate determination of likely disease progression before any treatment is begun. As some newer interventions in tetanus have been reported to reduce disease progression itself, this is especially important.^15–17^

In adult patients with tetanus, the efficacy of some interventions has been disputed due to potential differences in the severity of disease in the populations studied and the lack of consensus regarding how to quantify this.^18^ Some publications have used only overall mortality rates as a marker of disease severity.^19^ In neonatal tetanus, with wide variations in management and mortality rates ranging from 0 to 70% in different centres, improved indicators of disease severity are needed.^13,20–22^

In this study, we analyzed data from patients admitted with neonatal tetanus to our intensive care units. We examined multiple prognostic factors associated with outcome over a period of time during which improvements in critical care facilities were made.

## 2 Methods

All study patients were admitted to the Hospital for Tropical Diseases, Ho Chi Minh City, Vietnam between 1997 and 2012. The Hospital for Tropical Diseases is a tertiary referral hospital for infectious diseases and admits patients from the whole of southern Vietnam (population around 45 million). The reorganization of medical services in 2006 resulted in patients with neonatal tetanus being admitted to a specialist paediatric intensive care unit rather than the dedicated tetanus intensive care unit that had served all age groups and has been described previously.^23^ Facilities for the intensive care management of neonates available at the Hospital for Tropical Diseases include mechanical ventilation and invasive blood pressure monitoring. Over the course of the study, these facilities were gradually incorporated into standard neonatal tetanus management. Mechanical ventilation was first used in the management of neonatal tetanus in 2000. Invasive blood pressure monitoring was introduced in 2008 following the transfer of care to the specialist paediatric intensive care unit.

Two datasets were used for the analysis. For most analyses, data from both phases were combined. The first was a prospective collection of data from 87 consecutive neonatal tetanus cases admitted between 1997 and 2003 to the Tetanus Unit at the Hospital for Tropical Diseases as part of an ongoing tetanus surveillance programme.^23,24^ The second phase of the study consisted of case record analysis of 20 consecutive patients with neonatal tetanus admitted to the paediatric intensive care unit at the same hospital from January 2010 to December 2012.^25^

Clinical and demographic data were collected (Table 1). The incubation period was defined as the time between birth and first symptom, assuming umbilical portal of entry. The period of onset was defined as the time between first symptom and first generalized muscle spasm. Cases of ‘discharge to die at home’ or ‘discharge against medical advice’ were recorded as deaths, as these were felt certain to result in this outcome due to the severity of the disease. In phase 1 of the study, the results of routine neurological examinations performed on discharge were recorded, including screening for gross deficits and general development. Studies were approved by the scientific ethics committee of the Hospital for Tropical Diseases.

All data were entered into a specially designed database and extracted for analysis. Frequencies (%) and the median (with interquartile range, IQR) were used to describe data. Age and weight on admission were compared between phases of the study using the Wilcoxon rank sum test.

As mortality rates declined during the study period, we used a combined poor outcome endpoint of either death or staying in hospital for more than 40 days as a more sensitive means of detecting a poor outcome in a setting of low mortality. The cut-off of 40 days was based on the 75^th^ percentile of the length of hospital stay in patients who survived. Logistic regression was used for both univariate and multivariable analyses. Predefined covariates including age, clinical signs at admission (weight, temperature, heart rate, white blood cell count, and platelet count), and onset-related variables (time from first symptom to admission, incubation period, and the period of onset) were selected based on previous studies and clinical knowledge.^6,14,15^ Temperature, heart rate, and haematocrit on admission were not available for patients in phase 2 of the study. In the multivariable analyses, we excluded incubation period, as it was strongly related to age on admission but less reliable. We also included study period as a categorical variable, with three levels (1997–1999, 2000–2003, 2010–2012), in all univariate and multivariable analyses to adjust for changes in outcomes over time, because significant changes in management were made during these periods. As there was strong evidence for a non-linear effect of the time from first symptoms to admission on outcomes, we modelled separate linear effects for durations <6 days and ≥6 days, respectively. Univariate analyses were based on a complete-case analysis, excluding subjects with missing information for that variable. Multivariable analyses were based on multiple imputation of missing data. Effects of covariates on outcomes were summarized as the odds ratio (OR) with corresponding 95% confidence interval (95% CI). The following software was used in the analysis: IBM SPSS Statistics for Windows version 19.0 (IBM Corp., Armonk, NY, USA), R version 3.0.2 (R Foundation for Statistical Computing, Vienna, Austria), and the companion R package MICE version 2.21 for multiple imputation.^26^

## 3 Results

A total of 107 cases were included in the study: 87 in phase 1 and 20 in phase 2. Fifty-nine percent of patients were male. All except two patients were reported to be delivered at full term, with two patients having preterm delivery dates of 8 months and 8.5 months.

Admission characteristics are shown in Table 1. The median age on admission was 8 days (IQR 6–11 days) and median weight on admission was 2.8 kg (IQR 2.5–3 kg). These variables did not differ between the two study groups (*p*-values 0.847 and 0.353, respectively). The median time from first symptom to admission was 3 days (IQR 2–3.25 days). The median incubation period was 6 days (IQR 5–8 days), and the median period of onset was 24 h (IQR 24–24 h).

The overall mortality rate in the study was 40.2% (43/107). This changed significantly over time, falling from 58.7% (27/46) in years 1997–1999 to 31.7% (13/41) in 2000–2003, and finally to 15.0% (3/20) in 2010–2012 (likelihood ratio test, *p* < 0.001).

In contrast there was no clear evidence of change in the combined outcome measure of hospital stay >40 days or death, which was not significantly different between study periods: 63.0% (29/46), 51.2% (21/41), and 75% (15/20) for the same time intervals (likelihood ratio test, *p* = 0.179).

The analysis of admission factors in relation to the combined outcome measure and mortality is shown in Table 2. Younger age, reduced weight, shorter incubation period and period of onset, and increased white blood cell count on admission were all associated with increased mortality. In addition, a raised heart rate on admission was associated with the combined endpoint of death or prolonged hospital stay. The time from first symptoms to admission showed a pronounced non-linear effect on both outcomes (*p*-values for quadratic trend <0.01 for both outcomes) and therefore its effect was modelled as separate linear effects for durations <6 days and ≥6 days, respectively. In the univariate analysis, time from first symptom to admission was significantly associated with mortality (*p* < 0.001) and showed a U-shaped association: the risk of death decreased up to a period of 6 days, but increased again when the period was longer than 6 days. The risk of the combined endpoint also decreased significantly up to a period of 6 days (*p* < 0.001), but did not significantly change afterwards.

In the multiple logistic regression analysis of selected variables, shown in Table 3, weight and age were significantly associated with the composite endpoint: the OR for each kilogram increase in weight was 0.11 (95% CI 0.01–0.75) and the OR for each day increase in age was 0.73 (95% CI 0.56–0.95). Factors associated with mortality alone additionally included an increase in white blood cell count (OR for each 1000 cells increase 1.17, 95% CI 1.02–1.35), shorter period of onset (OR for each hour increase 0.94, 95% CI 0.88–0.99), and increased time from first symptom to admission when this was ≥6 days (OR for each day increase 3.77, 95% CI 1.14–12.51).

Twenty-six surviving patients in phase 1 of the study had a discharge assessment recorded. Overall 25/26 (96%) were considered to be developmentally normal for age at discharge, however 8/35 (23%) had residual muscle stiffness on discharge. Two patients returned for follow-up at 6 months and were examined by an experienced paediatrician. Both were judged developmentally normal for age.

## 4 Discussion

There are few reports of the outcome of neonatal tetanus in centres with facilities for mechanical ventilation and intensive care management. Jeena et al. reported outcomes and epidemiological factors in 27 cases of neonatal tetanus treated in a paediatric intensive care unit in South Africa in 1993, but did not analyze prognostic factors.^27^ We have previously shown in adults patients with tetanus that the prognosis is dependent on multiple variables.^10^ The recent meta-analysis by Lambo et al., which included 16 studies, examined only three variables: birth weight, age at onset, and age at presentation. They were unable to assess length of hospital stay and delay in hospitalization due to a lack of these data, but results showed that both age at presentation and birth weight were important predictors of mortality.^12^ The studies included in the meta-analysis were performed in Asia and Africa, spanning five decades, and included wide variations in management.

Despite the broader group of prognostic variables we examined, weight and age remained significant predictors of outcome. White blood cell count and heart rate were also associated with poor outcome in our series. This is consistent with previous findings in adults.^8,10^ However other factors also significant in adults, such as temperature on admission, were not found to be important markers of neonatal outcome. This may be due to our sample size or may reflect their relative unimportance in this age-group.

A delay in admission to hospital was associated with a worse outcome. For periods of time from first symptom to admission of ≥6 days, increasing time period was significantly associated with a poor outcome in the univariate analysis for both endpoints and remained significant in the multivariate model for mortality. Increased time from onset of symptoms to treatment may allow for increased toxin load or the development of secondary complications such as dehydration, sepsis, or aspiration pneumonia. The univariate analysis, however, showed that for shorter periods of time (<6 days) there was a reduced risk of a poor outcome with increasing time. This is likely to reflect the effect of rapid progression of severe disease, and in the multivariate model the effect was no longer significant when other variables such as age at presentation were included.

As the mortality rate declined significantly during the study period, we chose a combined endpoint of death or prolonged hospital stay to try and increase the sensitivity of our study. The combined endpoint measure did not show evidence of significant change over time and was associated with the same prognostic variables as mortality alone. Thus we believe this measure is an acceptable option when mortality rates are low and could be used to assess the efficacy of new interventions in settings such as ours. Indeed as the mortality rate changed significantly over time, the prognostic factors established as predictors of mortality may be influenced by the higher number of deaths in the earlier years of the study. Thus in choosing the most relevant prognostic factors to use clinically in future, those significant for both endpoints are perhaps the most important, i.e., age and weight.

We also note that a significant number of patients have hospital stays over 40 days. Previously some authors have defined neonatal tetanus mortality as ‘death within 28 days’.^12^ Three patients in phase 1 of this study, although no patients in phase 2, died after 28 days. Thus in centres where early deaths due to respiratory failure are avoided, such a definition is likely to underestimate true mortality.

In conclusion, the numbers of cases of neonatal tetanus admitted to our hospital over the last 15 years have fallen significantly and the outcome from the disease has improved. Prognostic factors previously identified in different settings, notably those with fewer resources, were still found to be associated with worse outcomes. In addition we have shown that a delay in admission to hospital and other factors associated with a worse prognosis in adults are also important in neonates in this setting. Survivors of neonatal tetanus require long periods of hospitalization and intensive care unit care, but little is known about long-term outcomes. Future research should be aimed at identifying cost-effective treatments able to reduce the duration of hospital stay as well as mortality, and at assessing long-term outcomes.

## Figures and Tables

**Table 1:** Clinical and laboratory features of participants on admission (*n* = 107)

Features	*n*	Median	(IQR)
Age, days	107	8.0	(6.0–11.0)
Time from first symptom to admission, days	104	3.0	(2.0–3.3)
Incubation period, days	105	6.0	(5.0–8.0)
Period of onset, h	102	24.0	(24.0–24.0)
Weight, kg	103	2.8	(2.5–3.0)
Temperature, °C	84	38.0	(38.0–39.0)
Heart rate, beats per min	85	140	(125–150)
White blood cell count, ×10^9^/l	98	12.0	(9.0–16.8)
Platelet count, ×10^9^/l	69	265.0	(190.0–444.0)
Haematocrit, %	67	47.0	(43.5–52.5)

IQR, interquartile range.

**Table 2:** Univariate effect of selected variables on mortality or length of hospital stay >40 days, and mortality only

		Mortality or LOS >40 days			Mortality		
Variable	*n*	OR	95% CI	*p*-Value	OR	95% CI	*p*-Value
Age (+1 day)	107	0.65	(0.52–0.77)	<0.001	0.68	(0.53–0.81)	<0.001
Weight (+1 kg)	103	0.09	(0.02–0.29)	<0.001	0.09	(0.02–0.32)	<0.001
First symptom to admission	104			<0.001			
+1 day, if <6 days^a^		0.49	(0.31–0.72)	<0.001	0.44	(0.26–0.69)	<0.001
+1 day, if ≥6 days^b^		1.60	(0.77–3.80)	0.212	4.29	(1.81–12.93)	<0.001
Incubation period (+1 day)	105	0.77	(0.66–0.88)	<0.001	0.79	(0.66–0.91)	<0.001
Period of onset (+1 h)	102	0.93	(0.89–0.96)	<0.001	0.94	(0.90–0.97)	<0.001
Temperature (+1 °C)	84	1.28	(0.82–2.03)	0.285	1.14	(0.73–1.81)	0.561
Heart rate (+10 beats per min)	85	1.37	(1.01–1.89)	0.040	1.1	(0.82–1.48)	0.526
White blood cell count (+1000/mm^3^)	98	1.10	(1.01–1.21)	0.025	1.09	(1.01–1.19)	0.024
Platelet count (+10 000/mm^3^)	69	1.00	(0.97–1.04)	0.864	1.01	(0.98–1.05)	0.568
Haematocrit (+1%)	67	1.04	(0.97–1.10)	0.262	1.04	(0.97–1.11)	0.249

LOS, length of hospital stay; OR, odds ratio; CI, confidence interval. All analyses were adjusted for time-period. Numbers in brackets after each variable indicate the required change in that variable to produce the described OR. As an example, the OR of 0.65 for age indicates that a 1-day increase in age results in a predicted reduction of the odds of the combined outcome of mortality or stay in the hospital for >40 days by 35%.

a The corresponding OR indicates the predicted change in the odds of the outcome for each increase in the time since first symptom to admission by +1 day as long as the durations is <6 days.

b The corresponding OR indicates the predicted change in the odds of the outcome for each increase in the time since first symptom to admission by +1 day for durations ≥6 days.

**Table 3:** Multivariable effect of selected variables on mortality or length of hospital stay >40 days, and mortality only (*n* = 107)

	Mortality or LOS >40 days			Mortality		
Variable	OR	95% CI	*p*-Value	OR	95% CI	*p*-Value
Age (+1 day)	0.73	(0.56–0.95)	0.021	0.69	(0.48–0.99)	0.042
Weight (+1 kg)	0.11	(0.01–0.75)	0.025	0.06	(0.01–0.54)	0.011
First symptom to admission						
+1 day, if <6 days^a^	0.77	(0.38–1.55)	0.466	0.72	(0.30–1.69)	0.446
+1 day, if ≥6 days^b^	1.13	(0.39–3.28)	0.817	3.77	(1.14–12.51)	0.029
Period of onset (+1 h)	0.94	(0.87–1.00)	0.067	0.94	(0.88–0.99)	0.046
Temperature (+1 °C)	1.45	(0.65–3.26)	0.364	1.40	(0.60–3.25)	0.431
Heart rate (+10 beats per min)	1.02	(0.97–1.08)	0.379	0.98	(0.92–1.03)	0.402
White blood cell count (+1000/mm^3^)	1.11	(0.96–1.27)	0.147	1.17	(1.02–1.35)	0.026
Platelet count (+10 000/mm^3^)	1.00	(0.99–1.00)	0.724	1.00	(0.99–1.01)	0.968
Haematocrit (+1%)	0.96	(0.85–1.07)	0.442	0.97	(0.84–1.11)	0.664
Study period						
1997–1999	1.00			1.00		
2000–2003	0.74	(0.13–4.06)	0.729	0.06	(0.01–0.41)	0.004
2010–2012	2.01	(0.19–20.58)	0.556	0.01	(0.001–0.17)	0.002

LOS, length of hospital stay; OR, odds ratio; CI, confidence interval. All missing data were imputed using multiple imputation. Numbers in brackets after each variable indicate the required change in that variable to produce the described OR. As an example, the OR of 0.73 for age indicates that a 1-day increase in age results in a predicted reduction of the odds of the combined outcome of mortality or stay in the hospital for >40 days by 27%.

a The corresponding OR indicates the predicted change in the odds of the outcome for each increase in the time since first symptom to admission by +1 day as long as the durations is <6 days.

b The corresponding OR indicates the predicted change in the odds of the outcome for each increase in the time since first symptom to admission by +1 day for durations ≥6 days.
